# Influence of epidermal growth factor receptor (EGFR), p53 and intrinsic MAP kinase pathway status of tumour cells on the antiproliferative effect of ZD1839 (‘Iressa’)

**DOI:** 10.1038/sj.bjc.6600299

**Published:** 2002-05-06

**Authors:** N Magné, J L Fischel, A Dubreuil, P Formento, M-F Poupon, P Laurent-Puig, G Milano

**Affiliations:** Department of Oncopharmacology, Centre Antoine Lacassagne, Nice, France; Institut Curie, Paris, France; INSERM U490, Paris, France

**Keywords:** ZD1839, EGFR, p53, MAPK, tyrosine kinase inhibitor

## Abstract

ZD1839 (‘Iressa’) is an orally active, selective epidermal growth factor receptor–tyrosine kinase inhibitor (EGFR–TKI), which blocks signal transduction pathways implicated in proliferation and survival of cancer cells, and other host-dependent processes promoting cancer growth. Permanent downstream activation of the mitogen-activated protein kinase pathway can theoretically bypass the upstream block of epidermal growth factor receptor-dependent mitogen-activated protein kinase activation at the epidermal growth factor receptor level. We investigated the impact of epidermal growth factor receptor content, p53 status and mitogen-activated protein kinase signalling status on ZD1839 sensitivity in a panel of human tumour cell lines: seven head and neck cancer cell lines and two colon cancer cell lines (LoVo, HT29) with derivatives differing only by a specific modification in p53 status (LoVo p53 wt + p53 mut cells, HT29 p53 mut + p53 wt rescued cells). The antiproliferative activity of ZD1839 was evaluated by the 3-(4,5-dimethylthiazol-2-yl)-2,5-diphenyl tetrazolium bromide test. ZD1839 concentrations ranged from 0.2–200 μM (48 h exposure). Epidermal growth factor receptor expression, p53 status and p42/p44 (for testing a constitutively active mitogen-activated protein kinase pathway status) were determined by competition analysis (Scatchard plots), denaturing gradient cell electrophoresis and Western blot, respectively. Epidermal growth factor receptor levels ranged from 388 to 33794 fmol mg^−1^ protein, a range that is similar to that found in head and neck tumours. The IC_50_ values for cell sensitivity to ZD1839 ranged from 6 to 31 μM and a significant inverse correlation (*P*=0.022, *r*=0.82) between IC_50_ values and epidermal growth factor receptor levels was observed. There was no influence of p53 status on the sensitivity to ZD1839. In two head and neck cancer cell lines with comparably elevated epidermal growth factor receptor expression, a two-fold higher ZD1839 IC_50_ value was found for the one with a constitutively active mitogen-activated protein kinase. In conclusion, ZD1839 was active against cells with a range of epidermal growth factor receptor levels, although more so in cells with higher epidermal growth factor receptor expression. Activity was unaffected by p53 status, but was reduced in cells strongly dependent on epidermal growth factor receptor signalling in the presence of an intrinsically activated mitogen-activated protein kinase pathway.

*British Journal of Cancer* (2002) **86**, 1518–1523. DOI: 10.1038/sj/bjc/6600299 www.bjcancer.com

© 2002 Cancer Research UK

## 

Epidermal growth factor receptor (EGFR), through binding to its physiological ligands, mainly epidermal growth factor (EGF) and transforming growth factor α (TGFα), is activated through autophosphorylation at defined cytoplasmic tyrosine residues. This facilitates association with several SH2 domain-containing proteins including PLC-γ, phosphoinositol-3-kinase, Grb2 and src family kinases. As a result of these interactions, signal transduction pathways are stimulated that can modulate several cellular key functions including proliferative activity and apoptosis (Chen *et al*, 1987; Schlessinger, 1994; Mercer, 2000; Schlessinger *et al*, 2000). One of the well studied intermediary steps concerns the activation of the proto-oncogene ras. The amplification of the erbB oncogenes, particularly the human EGFR gene *erbB-1* and ras proto-oncogene mutations, has been shown to play a fundamental role in the progression of different solid tumours, including head and neck cancer, by acting through the activation of the ras-MAPK (mitogen-activated protein kinase) pathway (Salomon *et al*, 1995; Maurizi *et al*, 1996; Grandis *et al*, 1996, 1998).

Epidermal growth factor receptor appears to be a major prognostic factor in head and neck cancer patients (Santini *et al*, 1991; Dassonville *et al*, 1993; Etienne *et al*, 1999). Thus, inhibition of EGFR represents a potential therapeutic strategy for controlling cancer growth, particularly in head and neck cancer where the expression of this receptor is frequently amplified. The development of compounds such as IMC-C225, an EGFR monoclonal antibody (Baselga, 2001), or ZD1839 (‘Iressa’), an orally active, selective EGFR tyrosine kinase inhibitor (Ciardiello and Tortora, 2001), is representative of this new therapeutic strategy for the treatment of cancer. The activity of trastuzumab (Herceptin) has been shown to be dependent upon Her 2 neu expression levels (Pegram *et al*, 1998, Pegram and Slamon, 2000) and it would therefore be interesting to determine whether or not the efficacy of EGFR targeting drugs is linked to EGFR tumour level.

Mutation or inactivation of ras and p53 oncogenes are the most common genetic events identified for almost all human cancers, including head and neck squamous cell carcinomas. In the context of drugs acting upstream to inhibit the initiation of MAPK pathway signal transduction, like IMC-C225 or ZD1839, it is interesting to note that the MAPK pathway can be constitutively activated by ras mutations (Hoshino *et al*, 1999). Changes in p53 status have been linked to disease progression, decreased sensitivity to chemotherapeutic agents and poor prognosis (Cabelguenne *et al*, 2000). The key role of p53 in controlling cell proliferation and apoptosis poses the question of whether p53 status influences the efficacy of EGFR signal inhibitors.

In this paper, we report the results of studies aimed to assess whether EGFR expression and intrinsic MAPK pathway status influences the cytostatic efficacy of EGFR signal inhibitors. We used a panel of seven human head and neck cell lines with a wide range of EGFR expression representative of that found in human tumours. In addition, the panel consisted of two pairs of cell lines, that differed in levels of MAPK activity and EGFR expression. In addition, a model of two human colon cancer cell lines, with specifically modified p53 status, was included to analyse the effect of p53 status on the efficacy of ZD1839.

## MATERIALS AND METHODS

### Chemicals

ZD1839 was kindly provided by AstraZeneca and a working solution (50 mM in dimethysulfoxide (DMSO)) was prepared immediately before use. Human recombinant ^125^I-EGF (ref. IM 196, specific activity 4514×10^10^ Bq/mol, 92.5×10^4^ Bq/250 μl) and unlabelled human recombinant EGF (ref. ARN 5100) were from Amersham. The agents were purchased: Dulbecco's modified eagle's medium (DMEM), Roswell Park Memorial Institute (RPMI 1640) and glutamine (Whittaker, Verviers, Belgium); foetal bovine serum (FBS) (Dutscher, Brumath, France); penicillin and streptomycin (Meyrieux, Lyons, France); transferrin and insulin (Flow, Irvine, Scotland); bovine serum albumin (BSA); phosphate-buffered saline (PBS); 3-(4,5-dimethylthiazol-2-yl)-2,5-diphenyl tetrazolium bromide (MTT) and DMSO (Sigma, St. Quentin Fallavier, France).

### Cell lines

A panel of seven head and neck cancer cell lines of human origin was specifically designed for this study: CAL27, CAL33, CAL60, CAL165, CAL166 originated from our Institute; Hep-2 and Detroit562 originated from the American Type Cell Collection (Rockville, MD, USA). Across all the cell lines, there was approximately a 100-fold difference in EGFR expression: Hep-2 expressed the lowest EGFR levels and CAL33 the highest (Table 1

**Table 1:**
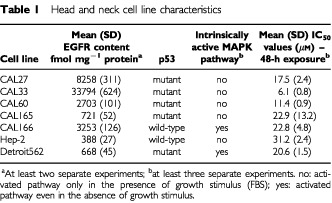
Head and neck cell line characteristics

). Two pairs of cell lines were represented in the panel: (1) CAL60 and CAL166, and (2) CAL165 and Detroit562. CAL60 and CAL166 both have high levels of EGFR expression whereas CAL165 and Detroit562 have relatively low levels. In addition, MAPK pathway status differed between cell lines in each pair: CAL166 and Detroit562 have a constitutionally active MAPK pathway whereas CAL60 and CAL165 do not.

Two groups of cell lines with specifically modified p53 status were also studied. These were obtained from Dr MF Poupon (CNRS, Institut Curie, Paris) and comprised: (1) LoVo cells (p53 wild-type) plus three derived cell lines (LoVo X2, LoVo X17, LoVo X19) in which mutated p53 was specifically introduced; (2) HT29 cells (p53 mutated) plus two derived cell lines (HT29 A3 and HT29 A4) in which p53 wild-type was re-introduced, as described by Soulié *et al* (1999). According to the findings of other authors (Pocard *et al*, 1996), LoVo was arrested in the G1 phase of the cell cycle after 10 Gy irradiation, while no arrest was obtained after irradiation of LoVo X2, LoVo X17, LoVo X19 with a mutated p53 status; HT29 A3 and HT29 A4 were arrested in the G1 phase after inhibition of pyrimidine synthesis, while no arrest was obtained with HT29 parental cells, showing the dominant function of wild-type p53 in the HT29 A3 and HT29 A4 cell lines.

Cells were routinely cultured in DMEM supplemented with 10% FBS, 2 mM glutamine, 600 μg l^−1^ insulin, 500 μg l^−1^ transferrin, 50 000 units l^−1^ penicillin and 80 μM streptomycin in a fully humidified incubator (Sanyo, Japan) at 37°C in an atmosphere containing 8% CO_2_.

### EGFR assay

Epidermal growth factor receptor expression was assayed as previously described (Olivier *et al*, 1990). Cells were grown to 80–90% confluence, in 24-well plates, in 10% FBS–DMEM at 37°C. Cells were rinsed three times with 500 μl RPMI 1640 containing 0.1% BSA at 2–4°C and incubated for 30 min with the same medium (500 μl per well) at 4°C. Total EGF binding was measured after incubation with 0.2 nM ^125^I-EGF (3 h, 4°C, 0.1% BSA–RPMI); non-specific binding was measured in the presence of an excess of unlabelled EGF (20 nM). The content of the EGFR was determined by incubation in RPMI medium for 3 h at 4°C in the presence of increasing concentrations of ^125^I-EGF (0.01, 0.02, 0.04, 0.08, 0.12, 0.18, 0.2 nM); for cells expressing high EGFR concentrations, cells were incubated with 0.2 nM of ^125^I-EGF with increasing concentrations of unlabelled EGF (0.05, 0.1, 0.2, 0.4, 0.8, 1.6, 3.2, 6.4, 20, 200 nM). Plates were put on ice to stop the reaction, the supernatant was removed, and cells were washed twice with PBS containing 0.1% BSA (4°C, 500 μl per well). Cells were solubilized in 1 M NaOH at 37°C (500 μl per well for 30 min). Radioactivity was determined by gamma counting. The number of receptor sites per cell (N) and the dissociation constant (Kd) were determined by Scatchard analysis (each point on the Scatchard plots was done in quadruplicate). Cell number was determined in four wells run in parallel, by resuspending cells in 200 μl PBS at room temperature and counting with a hemocytometer. Experiments were performed only in duplicate because of the intrinsic reproducibility of the assay (CV=7.3%, *n*=4).

### Determination of MAPK status

The determination of MAPK pathway status was based on the measurement of phosphorylated p42–p44 (activated form of MAPK) by immunoblot analysis performed in the presence (stimulated EGFR pathway, medium plus FBS) or absence of serum (non-stimulated EGFR pathway). No EGF was added. Prior to stimulation by growth factors, cells were grown in the presence of growth factors (i.e. FBS) for 48 h followed by a 48-h period in which they were deprived of growth factors (i.e. grown in serum-free medium): only control cells did not undergo growth factor deprivation and were grown in medium supplemented with FBS for 72 h. Stimulated (control) or non-stimulated cells (4×10^6^) were harvested in 50 μl of lysis buffer, Laemmli 1X (Laemmli 4X: 1.6 ml Tris-HCL 1 M pH 6.8; 400 mg sodium dodecyl sulphate (SDS); 2 ml glycerol; 145 μl β-mercaptoethanol; 12% bromophenol), and were heated for 15 min at 95°C. Protein content of the cytosol preparations was determined by the Bradford method using the Bio-Rad reagent with BSA as standard. Equal amounts of protein (50 μg/lane) were separated by 12.5% SDS–10% PAGE (polyacrylamide gel electrophoresis) and transferred onto a nitrocellulose membrane. Pre-stained molecular weight markers were included in each gel. Membranes were blocked for 30 min in tris-buffered saline (TBS)-Tween (10 mM Tris-HCl, pH 7.5; 150 mM NaCl with 0.5% Tween-20) and 5% BSA. After blocking, membranes were incubated for 12 h with a mouse anti-human monoclonal anti-MAP kinase activated antibody (Diphosphorylated ERK-1&2, anti-DPERK, clone MAPK-YT, dilution of 1/5000, Sigma) in TBS-Tween and 1% BSA. After washing the membranes three times with TBS-Tween (5 min each), they were incubated with peroxidase-conjugated secondary antibodies purchased from Dako (dilution of 1/1000, Glostrup, Denmark). The chemoluminescence reaction was performed and the membranes exposed to ECL hyperfilm according to the manufacturer's instructions (Amersham Pharmacia Biotech, Little Chalfont, UK). Triplicate determinations were made in separate experiments.

### Determination of p53 status

This specific investigation was performed by Dr P Laurent-Puig (INSERM U 490, Paris). DNA was first extracted and exons 4 to 8 were screened for mutations by denaturing gradient gel electrophoresis (DGGE) in accordance with the method described by Hamelin *et al* (1993) for exons 5, 7 and 8 and the method of Gulberg *et al* (1997) and colleagues for exons 4 and 6. Exon 9 was screened for mutations by the method described by Cabelguenne *et al* (2000). PCR amplification products were loaded onto a 6.5% polyacrylamide gel that contained an appropriate gradient of urea and formamide. After electrophoresis, gels were stained with ethidium bromide. Tumours that showed an electrophoresis variant pattern were amplified and sequenced for each variant exon. PCR products were purified with QIAquick PCR Purification Kit (QIAGEN S.A., Courtabeuf, France) and sequenced on both strands on an ABI 310 genetic analyser (PE Applied Biosystems, Courtabeuf, France). A Big Dye Terminator sequencing kit (PE Applied Biosystems) was used according to the manufacturer's instructions, followed by ethanol precipitation, to remove nonincorporated dyes. Sequences were analysed by Sequence Analysis 3.0 (PE Applied Biosystems).

### Evaluation of ZD1839 antiproliferative activity

Cells were seeded in 96-well microtitre plates (100 μl per well) and incubated for 48-h to establish exponential growth (initial cell density was 2000 cells/well for CAL165; 2500 cells/well for CAL27 and Hep-2; 3000 cells/well for CAL33, Detroit562, LoVo and HT29; 4500 cells/well for CAL166; 5000 cells/well for CAL60). Cells were then incubated with ZD1839 (0.2–200 μM) for 48 h; eleven concentrations were tested for each cell line.

Growth inhibition was assessed by the MTT test (described below (Carmichael *et al*, 1987)) 48 h after ZD1839 was removed. Cells were washed with PBS and incubated with MTT for 2 h, followed by the addition of 100 μl of DMSO. Absorbance at 450 nm was measured using a microplate reader (Labsystems, Helsinki, Finland) and results were expressed as the relative percentage of absorbance compared with controls. Experimental conditions were tested in sextuplicate (six wells of the 96-well plate per experimental condition), and experiments were performed in triplicate. The dose-effect curves were analysed on Prism software (GraphPad Software, San Diego, USA). The antiproliferative activity of ZD1839 was expressed by the IC_50_ value (concentration leading to 50% reduction in cell growth measured by MTT).

### Statistical analysis

The relationship between EGFR expression and response to ZD1839 was analysed by plotting the IC_50_ of ZD1839 sensitivity for cell lines *vs* EGFR content. The correlation coefficients (*r*) and the *P* values (*P*<0.05 was considered statistically significant) were computed using the program SPSS software (Chigago, IL, USA).

## RESULTS

### Cell line characteristics

Cell line characteristics are displayed in Table 1. The assessment of EGFR content confirmed that EGFR expression varied substantially between cell lines in the panel of head and neck human squamous cell carcinoma cells. In addition, five of the seven human head and neck cell lines were confirmed as having a mutated p53 gene: CAL166 and Hep-2 were p53 wild-type. Two cell lines, CAL166 and Detroit562, had a constitutively activated MAPK pathway.

The EGFR content in LoVo and HT29 cell lines (parental and transfected) was 65 to 78 fmol mg^−1^ protein and 3293 to 3450 fmol mg^−1^ protein, respectively (Table 2

**Table 2:**
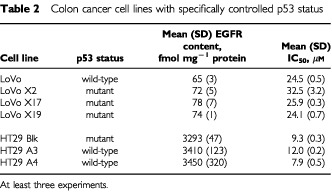
Colon cancer cell lines with specifically controlled p53 status

).

### Antiproliferative effect of ZD1839 on head and neck cell lines

Figure 1

**Figure 1:**
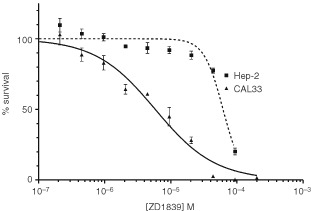
Typical dose–response curves for ZD1839 on CAL33 and Hep-2 cell lines.

shows typical dose–response curves for ZD1839 on CAL33 and Hep-2 cell lines. IC_50_ values and other characteristics for the head and neck cell lines tested are presented in Table 1. CAL33 cells were found to be the most sensitive to the growth inhibitory effects of ZD1839 (IC_50_ 6.1 μM), and Hep-2 cells were the least sensitive (IC_50_ 31.2 μM).

### Relationship between EGFR expression and response to ZD1839

**Figure 2:**
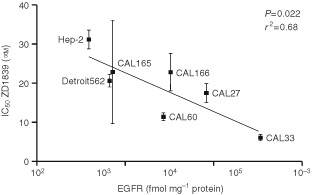
Link between cell sensitivity to ZD1839 and EGFR content (48-h exposure).

A significant inverse correlation was found between ZD1839 IC_50_ value and EGFR content (*P*=0.022, *r*=0.68) (Figure 2

). The inclusion of LoVo and HT29 cell lines in the regression analysis improved the correlation with an *r* shifting from 0.68 to 0.72 and the *P* value from 0.022 to 0.004 (not shown).

### Influence of p53 status on cell sensitivity to ZD1839

Cell sensitivity to the growth inhibitory effects of ZD1839 remained unchanged irrespective of p53 status (Table 2). This was also the case when p53 wild-type was introduced in a p53 mutant cell line (HT29 series) and when a p53 mutant was transfected in a p53 wild-type cell line (LoVo series). Overall, HT29 cells were more sensitive than LoVo cells to the effects of ZD1839; this could be attributable to the difference in EGFR content between HT29 cells and LoVo cells (Table 2).

### Influence of MAPK pathway status on cell sensitivity to ZD1839

Two pairs of cell lines having comparable EGFR levels but with different MAPK pathway status have been compared (CAL60/CAL166 and CAL165/Detroit562). Within each pair, the difference of sensitivity to ZD1839 due to the status of MAPK pathway depended upon whether the cells had a high or low EGFR content: for the pair with a high EGFR content, CAL60 (MAPK pathway not intrinsically active) was found to be twice as sensitive to ZD1839 as CAL166 (MAPK pathway intrinsically active) with respective IC_50_ values of 11.4 and 22.8 μM, whereas for the pair with low EGFR content, there was no difference in sensitivity between CAL165 and Detroit562 (Table 1).

## DISCUSSION

Overexpression of EGFR predicts a poor prognosis in many cancers, particularly in head and neck squamous cell carcinomas (Santini *et al*, 1991; Dassonville *et al*, 1993; Salomon *et al*, 1995; Maurizi *et al*, 1996; Grandis *et al*, 1996, 1998; Etienne *et al*, 1999), and consequently EGFR is a prime target for anticancer therapy. For monoclonal antibodies targeting EGFR, it has recently been shown that high expression of EGFR is associated with a better efficacy of the biologic agent (Hambek *et al*, 2001). Other investigators (Bos *et al*, 1997) have also provided evidence for a link between EGFR level and the sensitivity to a EGFR tyrosine kinase inhibitor; but the debate is still open (Moasser *et al*, 2001) and data based on a sufficient number of cell lines with a quantitative determination of EGFR cellular level are still justified. We thus examined the relationship between EGFR content of cells across a representative panel of head and neck cell lines and cell sensitivity to ZD1839. The present data establish a strong correlation between cell sensitivity of ZD1839 and EGFR expression, with the highest ZD1839 efficacy in cells with the highest EGFR content, although the clinical significance of this is unknown. The IC_50_ values reported here are substantially higher than those published by other investigators (Ciardiello *et al*, 2000). The used drug concentrations were dictated by the experimental conditions and, as compared to *in vivo* conditions, to a relatively short exposure of the tumoural cells to the drug (given once). It is normal that, *in vivo*, when tumour cells are continuously exposed to the parent drug, the observed concentrations may be lower; *in vivo* there is also the possible presence of active metabolites carrying a part of the activity, such metabolites are obviously not present in the *in vitro* condition. On the other hand, for cell survival *in vitro*, the presence of a percentage of serum in the culture medium is needed and this situation brings growth factors which stimulate the drug pathway which is targeted by the drug. Other investigators using similar experimental conditions than ours have recently reported on ZD1839 IC_50_ values very close to those reported in this manuscript (Al-Hazzaa *et al*, 2001). To match *in vivo* conditions as closely as possible, we have deliberately not used serum-free conditions. In additionnal experiments (data not shown), cell lines have been exposed to a medium supplemented with TGFα (1.10^−3^–10 ng ml^−1^) and EGF (1.10^−3^–10 ng ml^−1^); no differences in the IC_50_ values of ZD1839 were observed in any tested cell line. This suggests that EGFR ligands in the serum-containing medium were sufficient to saturate EGFR. The present data show a positive correlation between cell sensitivity of ZD1839 and EGFR expression. Despite this correlation, antiproliferative activity of ZD1839 continued to be seen at low levels of EGFR expression. The response to EGFR-targeted agents may be considered as the net outcome of a multifactorial set of circumstances; EGFR number *per se* may only be one component of this, as exemplified by the data presented here on the influence of constitutively activated MAPK on response.

Previous data have shown that ZD1839 has numerous effects on tumour cells including cell cycle arrest, increase in apoptosis and reduction in cell proliferation (Ciardiello *et al*, 2000; Sirotnak *et al*, 2000). It is clear that p53 status and EGFR signalling must interfere at the level of cell cycle control and apoptosis. Thus, data examining the role of p53 in the efficacy of EGFR targeting (with an adequate cellular model controlling p53 status only) need to be provided. Two recent papers suggest an interaction between EGFR and p53 (Donato *et al*, 2000; Akerman *et al*, 2001). As a consequence of this, analysis of the possible influence of p53 mutation and intrinsic MAPK pathway status was an additional objective of the present study. In head and neck squamous cell carcinomas the pivotal role of the tumour suppressor gene p53 in determining the balance between cell proliferation and cell death is well established (Mineta *et al*, 1998; Temam *et al*, 2000). In the present panel of seven head and neck cell lines, the high proportion of cell lines mutated for p53 concurs with the mutational status of p53 in oral carcinomas and supports the clinical relevance of the experimental model used in this study. However, this head and neck cell line panel did not permit a clear conclusion on whether p53 status impacts on ZD1839 sensitivity, due to the fact that other confounding cellular factors, such as EGFR content, also influence drug response. Thus, an additional series of two human colorectal cancer cell lines (LoVo and HT29) was added. These cell lines constitute an appropriate model for specifically testing the impact of p53 status because they differ only with respect to their genetic alterations concerning p53. Clearly, on this experimental basis, the cellular p53 status did not influence ZD1839 efficacy. On the other hand, it has been recently shown that *in vitro* manipulations of p53 status could influence EGFR expression level (Ludes-Meyers *et al*, 1996). In the present study there was no influence of p53 status on EGFR content. This observation was not only true for a situation where wild-type p53 was transfected in a p53 mutant cell line, but also for the opposite situation, where p53 mutant was transfected in a wild-type p53 cell line.

In contrast with the experiments testing the effect of p53 status, the situation for MAPK pathway status depended upon the model used. It is well known that ras mutations lead to a constitutionally active MAPK pathway, but the inverse is not necessarily true, and spontaneous phosphorylation of p42/p44 can also be encountered in tumours with intact ras (Hoshino *et al*, 1999; Bar-Sagi and Hall, 2000). Thus, in the present panel, cells with an intrinsically active MAPK pathway were not to be *a priori* considered as ras mutated. Two pairs of cell lines having, for each pair, similar EGFR content and different intrinsic MAPK pathway status were at our disposal in the panel: one pair with relatively high EGFR levels (CAL60/CAL166) exhibiting a difference in sensitivity to ZD1839 between each cell line of the pair, and the other pair of cell lines with relatively low EGFR levels (CAL165/Detroit562) showing no difference in sensitivity to ZD1839 between each cell line of the pair. A key finding from these results was that even in the presence of a constitutively active EGFR–MAPK pathway, which could theoretically bypass an upstream signal blockade, the application of the EGFR–TKI ZD1839 could still lead to the abrogation of cell proliferation. In addition, it was shown that MAPK pathway status may influence ZD1839 activity only in tumour cells that are strongly dependent upon EGFR signalling (high EGFR content). We are conscious that these results are preliminary in nature but they suggest that an intrinsically activated MAPK pathway could represent a source of intrinsic resistance to ZD1839.
